# Exploration and comparison of molecular mechanisms across diseases using MINERVA Net

**DOI:** 10.1002/pro.4565

**Published:** 2023-02-01

**Authors:** Piotr Gawron, Ewa Smula, Reinhard Schneider, Marek Ostaszewski

**Affiliations:** ^1^ LCSB, Luxembourg Centre for Systems Biomedicine University of Luxembourg Esch‐sur‐Alzette Luxembourg

**Keywords:** disease maps, network analysis, protein interactions, systems biology

## Abstract

Protein function is often interpreted using molecular interaction diagrams, encoding roles a given protein plays in various molecular mechanisms. Information about disease‐related mechanisms can be inferred from disease maps, knowledge repositories containing manually constructed systems biology diagrams. Disease maps hosted on the Molecular Interaction Network VisuAlization (MINERVA) Platform are individually accessible through a REST API interface of each instance, making it challenging to systematically explore their contents. To address this challenge, we introduce the MINERVA Net web service, a repository of open‐access disease maps allowing users to publicly share minimal information about their maps. The MINERVA Net repository provides REST API endpoints of particular disease maps, which then can be individually queried for content. In this article, we describe the concept of MINERVA Net and illustrate its use by comparing proteins and their interactions in three different disease maps.

## 1 INTRODUCTION

In order to research mechanisms underlying human diseases we need a better understanding of the interconnections between key molecules implicated in a given pathology. Our knowledge about these molecular mechanisms grows and is consistently captured by publicly available pathway databases (Gillespie et al., 2022; UniProt Consortium, 2021), collections of protein interaction networks (Lo Surdo et al., 2018; Perfetto et al., 2020), and disease maps (Mazein et al., 2018). Disease maps are resources that are usually constructed independently from main pathway and interaction databases, relying on dedicated software to host and share their content. The Molecular Interaction Network VisuAlization (MINERVA) Platform is a software widely used to visualize and analyze disease maps (Gawron et al., 2016; Hoksza et al., 2019), offering extensive API connected to a backend database, supporting reproducible research workflows.

Building and use of disease maps are a decentralized effort, making it challenging to inform other researchers about ongoing or completed work. This is a drawback in comparison to centrally managed databases, where content can be accessed and explored using a uniform querying scheme. Providing such functionality is important to support community‐driven knowledge building and exchange. Moreover, sharing information across disease maps will allow investigating multiple diseases at the same time, providing insight into comorbidities and specifics of different pathologies (Glaab & Schneider, 2015).

Here, we introduce MINERVA Net, a registry of publicly shared disease maps projects. Establishing such a registry is possible due to the development of the MINERVA Platform, which now enables users to share information about their projects on the central server of MINERVA Net. This information, available via dedicated API, can be used to query individual instances of the MINERVA Platform using their API interfaces, to explore and compare the contents of different disease maps. In the remainder of the paper, we introduce new functionalities of the MINERVA Platform, the newly established MINERVA Net registry as well as a simple R package minervar for better streamlining of computational workflows. Using this setup, we compare contents and protein interaction networks across three disease maps covering different aspects of age‐related neurodegeneration.

MINERVA Net registry is available at https://minerva-net.lcsb.uni.lu/.

R package minervar is available at https://gitlab.lcsb.uni.lu/minerva/minervar.

R code to run the discussed examples is available at https://gitlab.lcsb.uni.lu/minerva/api-scripts/

## 2 METHODS

### 2.1 The MINERVA Platform

**FIGURE 1:**
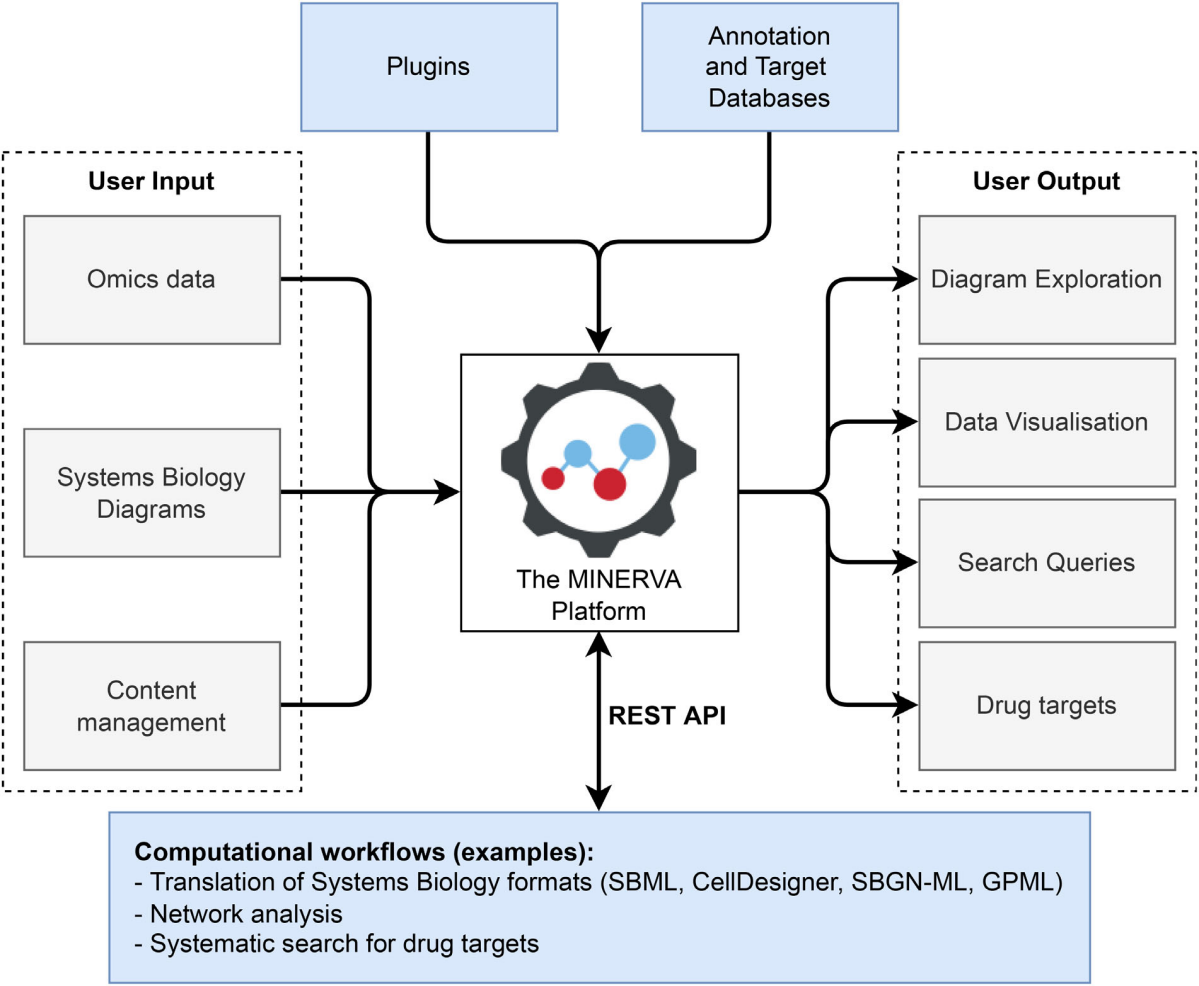
Summary of functionalities of the Molecular Interaction Network VisuAlization (MINERVA) Platform. Each instance of the Platform offers web‐based upload and management of user‐provided diagrams in various standard formats. These diagrams are enriched with contents of annotation and target databases and can be explored online together with associated omics data. A dedicated plugin architecture and REpresentational State Transfer Application Programming Interface (REST API) provide a computational interface for custom visualization and analytical workflows.

The MINERVA Platform (Gawron et al., 2016) is a standalone web server for visualization of molecular interaction diagrams (https://minerva.uni.lu). The instances of MINERVA Platform are independent from each other and can be deployed on a dedicated IT infrastructure. Figure 1 summarizes the functionalities of the MINERVA platform.

The MINERVA Platform can host diagrams in different systems biology formats (Hoksza et al., 2021) and handle their annotation in a uniform way. Importantly standard protein identifiers are supported, together with visualizing protein structures and their annotations using MolArt software (Hoksza et al., 2018). Taken together, the Platform supports visual exploration and research of protein‐level information, also in the context of human diseases. However, in case of extensive disease maps, manual exploration of proteins of interest, their network neighbors or drug targets, can be time‐consuming and error‐prone. To address this issue, the users can encode their visual exploration routines into reproducible workflows relying on an extensive REpresentational State Transfer Application Programming Interface (REST API) featured by the MINERVA Platform (Hoksza et al., 2019). The REST API allows retrieving information about diagram interactions and their elements, including annotation, coordinates, drug‐targeting molecules, and network connectivity.

An important feature of the Platform is the management of uploaded diagrams, including control over access rights. Setting different visibility and editing privileges allows a single MINERVA instance to host multiple projects handled by different curators. Newly introduced extension (version 16.1, see https://minerva.pages.uni.lu/doc/releasenotes) allows platform administrators to register a MINERVA Platform instance in the MINERVA Net registry. This in turn allows the curators of diagrams hosted on a given MINERVA instance to share information about their projects via the registry, using a simple GUI functionality. Only a minimal set of information about the MINERVA Platform instance, and about the shared projects, is deposited in MINERVA Net, see Table 1.

**TABLE 1:** Information sent to the MINERVA Net registry.

Registration of	Parameter	Visibility
MINERVA Platform instance	The URL of this instance	Public
	Email of the platform administrator	Internal
Project	Name, identifier, and version	Public
	Timestamps of project creation and sharing	Public
	Associated disease and organism	Public
	Email of the project curator	Internal

*Note*: Public information will be shared via the repository. Internal information will be used for service maintenance and communication with the administrators and curators using a given instance of the MINERVA Platform.

### 2.2 MINERVA Net registry

**FIGURE 2:**
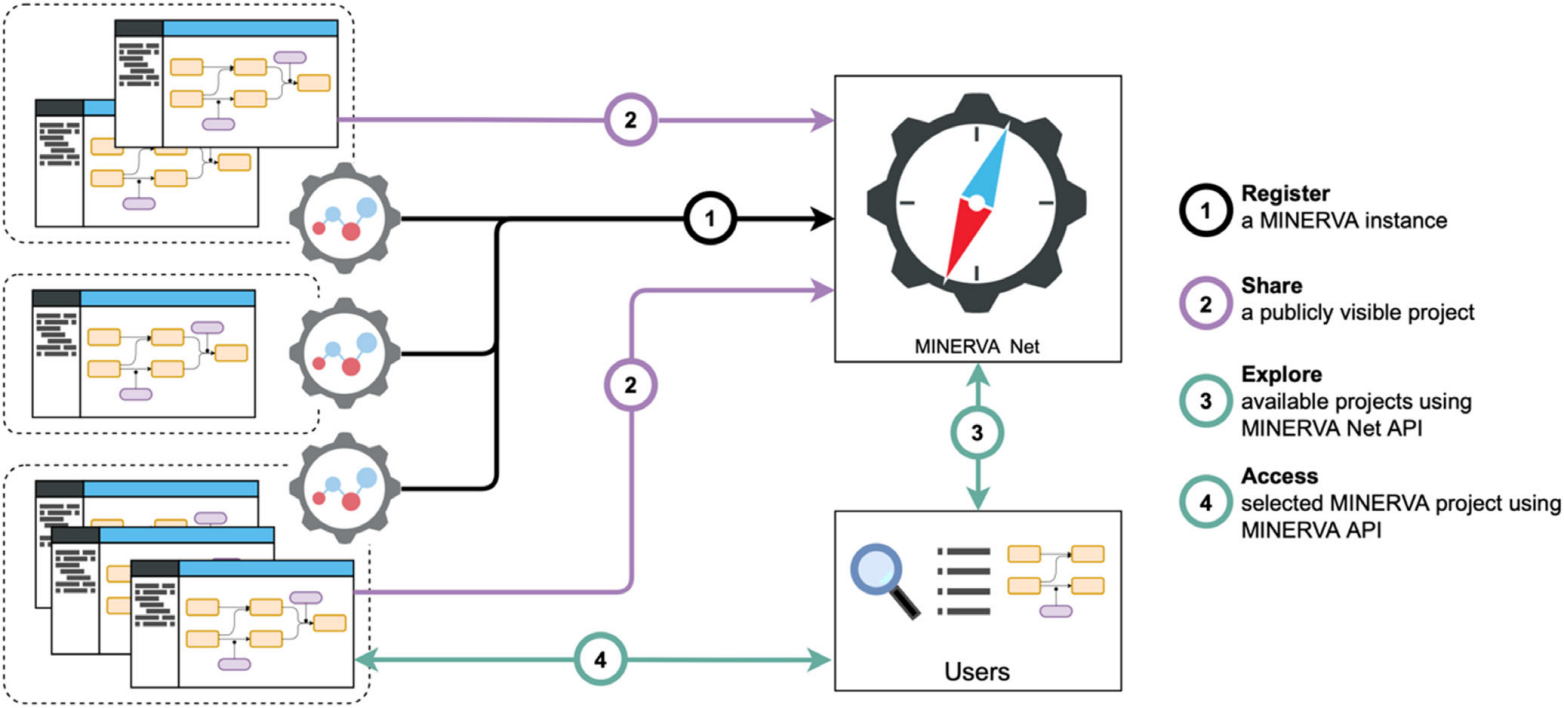
Molecular Interaction Network VisuAlization (MINERVA) Net registry. Instances of the MINERVA Platform are registered in MINERVA Net, to share information about publicly visible projects on the registered instances. Using the API of MINERVA Net, these projects can be browsed and accessed using the API of the given instance.

MINERVA Net (https://minerva-net.lcsb.uni.lu) is an API‐based service allowing users to register an instance of MINERVA Platform, and then to serve information about projects shared on this registered instance (see Figure 2). MINERVA Net API calls used for registration are paired with the MINERVA Platform functionality (see earlier Section) introduced as a wrapper for these calls. This way, the communication of MINERVA Net with different instances is supported by a user interface.

When registering an instance, MINERVA Net collects the URL of this instance and the email of the platform administrator (see Table 1). The email is used only internally, to inform about potential communication errors observed by the registry, related to the availability of the instance. When an instance is registered, sharing of projects becomes possible. When registering a project, MINERVA Net collects (i) name, identifier, and version, (ii) timestamps of project creation and sharing, and (iii) associated disease (MeSH identifier) and organism (NCBI Taxonomy ID). This information becomes available via the MINERVA Net API. Additionally, the registry collects the email address associated with the project to inform about potential communication errors observed by the registry, related to the availability of the project. Importantly, only projects publicly visible on a given instance of the MINERVA Platform can be shared via the registry. This way the registry contains information that is otherwise openly accessible.

This setup allows building workflows exploring multiple projects across different instances of MINERVA Platform. Users can use MINERVA Net API calls to read and filter projects of interest available on the registry. They can then retrieve addresses of particular projects and use MINERVA Platform API to query the contents of the projects, which includes element annotation, network connectivity, or graphical information.

### 2.3 R package minervar

To facilitate communication with the MINERVA Platform and MINERVA Net registry, we developed an R package minervar (https://gitlab.lcsb.uni.lu/minerva/minervar). The package is a wrapper to both APIs focusing on retrieving (i) project information, (ii) elements, reactions, and annotations of hosted diagrams, and (iii) MINERVA Net registry contents. It also handles map comparison tasks discussed in this article. Other functionalities of MINERVA Platform API, like retrieving drug targets or modifying project contents, are currently not covered.

### 2.4 Data and analysis

The analysis was performed using the MINERVA Net registry version 1.0.2, MINERVA Platform version 16.2.7, and minervar package version 0.8. All code is available in a public repository: https://gitlab.lcsb.uni.lu/minerva.

Analysis was performed in R and is available in a public repository: https://gitlab.lcsb.uni.lu/minerva/api-scripts/.

## 3 RESULTS

### 3.1 Comparing disease maps using MINERVA Net

To demonstrate the use of MINERVA Net registry in discovering the role of proteins and their interactions, three disease maps were compared focused on neurodegeneration and aging, namely Parkinson's disease map (Fujita et al., 2014), AlzPathway (Ogishima et al., 2016) and Aging map focused on mechanisms of progeria (https://progeria.uni.lu). To this end, we filtered the three most recent entries from MINERVA Net with MeSH identifiers D010300, D000544, and D008659.

To investigate common mechanisms between these disorders we focused on stable protein identifiers, as they participate in both signaling and metabolic pathways. For this purpose, we collected UniProt identifiers (UniProt Consortium, 2021) (https://www.uniprot.org) from selected disease maps. We decided to discard the information about protein active forms or their posttranslational modifications, otherwise available in the systems biology supported by the MINERVA Platform, as this was beyond the scope of this article. Nevertheless, this information can support future comparison approaches.

### 3.2 Comparison of content

We compared pairwise protein identifiers in diagrams of the selected disease maps. To this end, we queried the MINERVA Platform instances for elements and their annotations and retrieved available UniProt entries. Out of 515 entries common for at least two maps, the most overlap is shown between the Aging and Parkinson's maps (426 entries). In turn, Aging and AlzPath (94 entries), and AlzPath and Parkinson's map (161 entries) have markedly fewer common proteins. Table 2 summarizes the overall numbers of similar proteins shared between the maps, including a Jaccard similarity score ($\frac{\left|A\cap B\right|}{\left|A\cup B\right|}$) for each pairwise comparison or protein sets (see Table 3).

**TABLE 2:** Disease maps selected for comparison.

Map name	MeSH ID	Nb of diagrams	Nb of elements	Nb of interactions	Nb of unique UniProt IDs
Parkinson's disease map	D010300	22	9319	4170	1370
AlzPathway	D000544	1	2487	982	615
Aging map	D008659	5	3085	1103	360

*Note*: Summary information.

**TABLE 3:** Similarity of proteins between diagrams in the selected disease maps.

	Parkinson's map	Aging map	AlzPathway
Parkinson's map	1370 proteins	423 similar	161 similar
Aging map	Jaccard: 0.271	615 proteins	94 similar
AlzPathway	Jaccard: 0.103	Jaccard: 0.107	360 proteins

*Note*: Upper triangle: number of unique proteins shared between the maps; diagonal: number of unique proteins in a given map; lower triangle: Jaccard similarity between sets of unique proteins.

**FIGURE 3:**
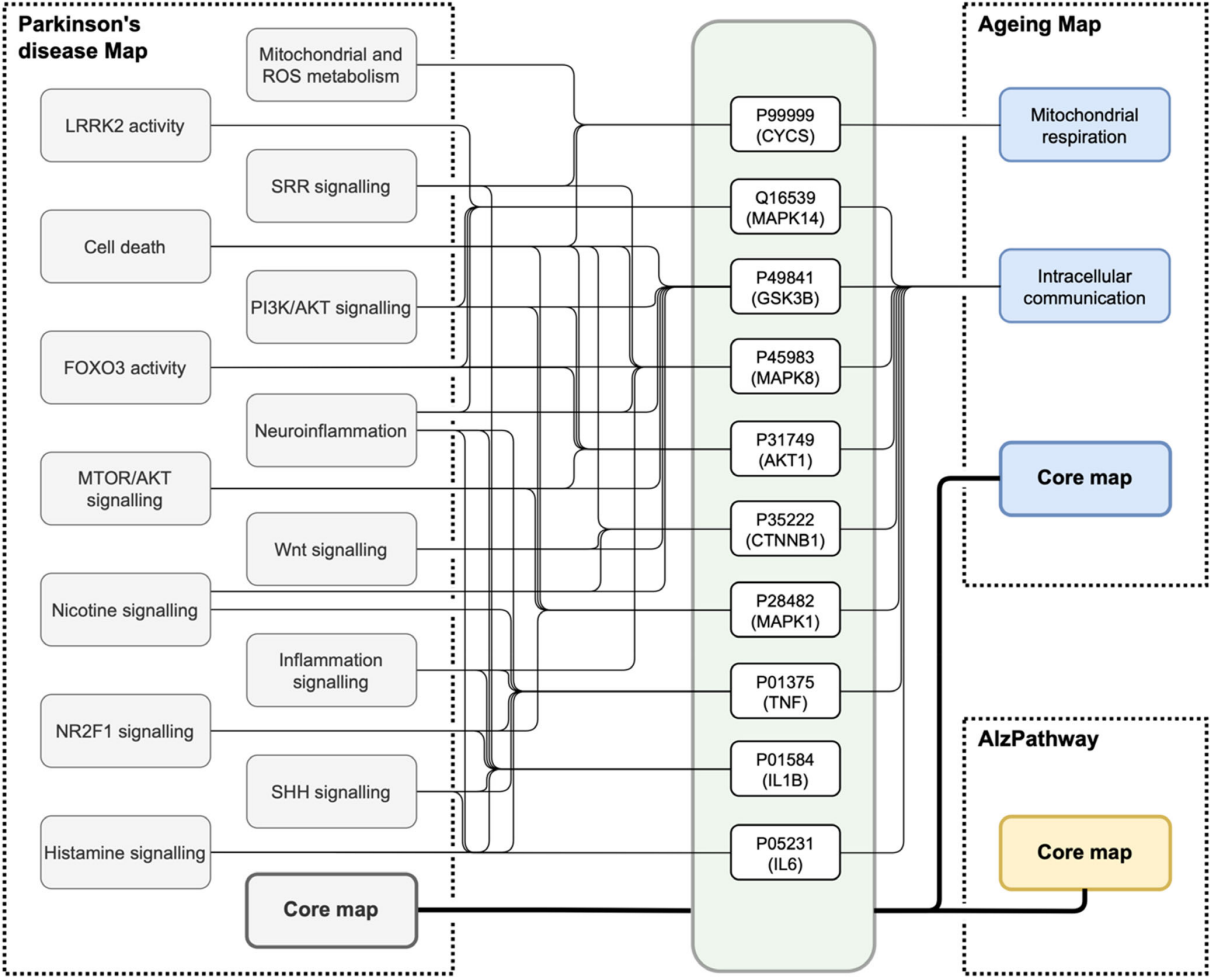
Proteins frequently shared between diagrams in the selected Maps. Ten proteins most frequently shared between three Maps. In each Map, “Core map” refers to the main diagram, and remaining elements are named after corresponding diagrams. All of the indicated proteins are in the main diagram of each Map. AlzPathway is composed of a single diagram.

The results for the 10 most interconnected proteins are illustrated in Figure 3. Interestingly, the processes implicating these proteins range from basic cell signaling (MAPK and AKT1), through components of immune response (TNF, ILB, and IL6) to neuronal pathways (GSK3B and CTNNB1). The full table is available in Appendix S1.

These results follow the line of recent research indicating involvement of mitochondrial pathways (Green et al., 2011) and chronic inflammatory processes in aging (Wyss‐Coray, 2016). In turn, the MAPK signaling molecules are represented in different contexts across the maps. In AlzPathway, they are a part of a generic MAPK signaling module and are involved in cell death signaling and APP related mechanisms. In the Aging map, MAPK molecules are a part of cell cycle and survival mechanisms of non‐neuronal cells, and neurotoxicity‐related signaling. Interestingly, the overlap between the PD map and AlzPathway does not involve microglial mechanisms, which emerge as an important factor in both neurodegenerative diseases (Paolicelli et al., 2022). The reason for this may be that the content of AlzPathway was not recently updated, indicating the need for a revision.

### 3.3 Similarity of interactions

We wanted to investigate the overlaps between the disease maps with more detail. For this reason, we retrieved information about element interactions from the MINERVA Platform instances. We treated each interaction as a list of elements, discarding the available information about the role of each element (reactant, product or modifier). As in Section 3.2, we retrieved UniProt identifiers for elements in interactions, ignoring elements without available identifiers. Importantly, we kept repeating UniProt identifiers, which can appear for instance in case of homodimer complexes, or reactions having different posttranslationally modified versions of the same protein. With this, we calculated the similarity between such lists of identifiers using the following formula:

$$\text{Similarity}\;\left(A,B\right)=\frac{n\left(A\circ B\right)*n\left(B\circ A\right)}{n\left(A\right)*n\left(B\right)},$$

where *A* and *B* are lists of UniProt identifiers, *n*(*A*) is the number of elements in *A*, and ∘ is an operator of list intersection with duplicates. Because lists *A* and *B* can contain repeated identifiers, A∘B≠B∘A.

We also counted the absolute number of proteins overlapping in both interactions. These parameters (similarity, number of overlapping identifiers in *A* and in *B*) were collected for all pairwise comparisons between interactions of selected disease maps. We focused only on interactions having similarity of 0.7 or more and having at least three overlapping identifiers in both interactions. The full table of these results is available in Appendix S1.

Following the comparison of the overall protein coverage, the overlap between the Aging map and AlzPathway is markedly smaller, accounting to 34 comparisons meeting the filtering criteria, while the Parkinson's disease map and AlzPathway have 43 similar interactions. In turn, the Parkinson's disease map overlapped with the Aging map in 342 cases. Such a reaction‐wise comparison allows informative visual exploration of areas between disease maps that cover similar molecular mechanisms. Identification of such areas is more informative than lists of similar proteins. For instance, comparison of common interactions between the PD map and the Aging map reveals large overlaps in mechanisms implicated in protein degradation (Autophagy, Ubiquitin‐proteasome system), mitochondrial biogenesis and quality control, and mTOR/AKT signaling. Interestingly, progeria‐specific mechanisms in the Aging map, involving SREBF1, GSK3A/B, and FBXW7, are related to PGC1A activity in the PD map, indicating potential cell and tissue specificity of the mechanism. The comparison of AlzPathway with the Aging map indicates only two clusters of interactions, around protein degradation and AGER signaling, related to the amyloid pathway (Hampel et al., 2021). Finally, similar reactions between the PD map and AlzPathway mostly group around protein degradation and inflammation signaling pathways.

**FIGURE 4:**
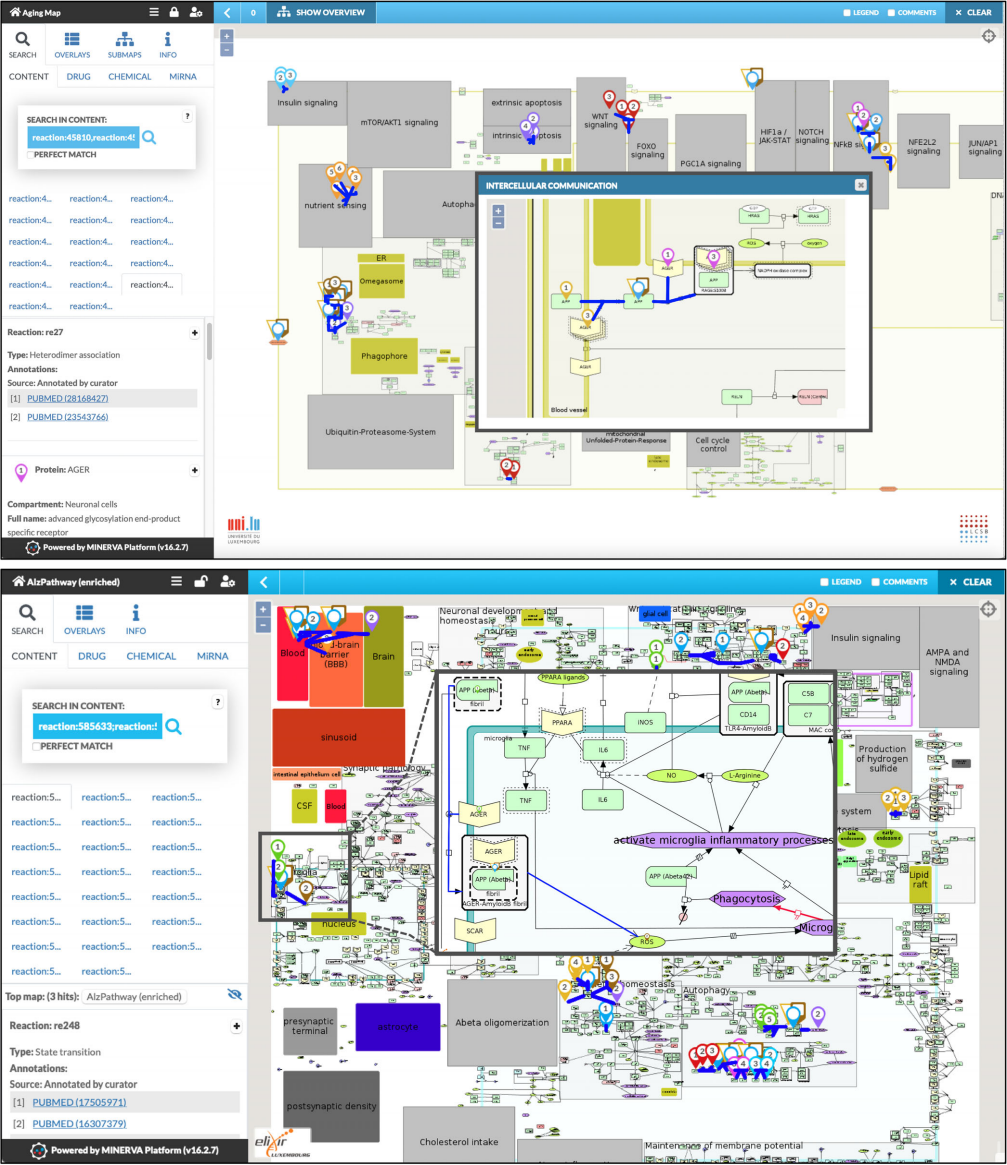
Visualization of protein interactions having high similarity between two disease maps. The top part is the Aging map, the bottom is AlzPathway, and both maps are shown in the Molecular Interaction Network VisuAlization (MINERVA) Platform interface. Interactions similar between these two maps (see text) are highlighted in blue, components of these interactions are marked as colored pins. AGER signaling interactions are highlighted (i) in the Aging map (top) as a zoomed in submap and (ii) in the AlzPathway as a zoomed in inset.

To illustrate how such a comparison looks like, we visualized map interactions similar between Aging map and AlzPathway, using an example with a manageable number of such interactions. Figure 4 shows the interactions in the two maps side‐by‐side, highlighted in the MINERVA Platform. In the case of Aging map, results from the “Intercellular communication” are shown as well. In this Figure, all common interactions are visible, and can be further explored using pan and zoom functionalities. In the example, most of the interactions were found in the main diagram, with one in the “Intracellular signaling”, demonstrating the APP‐AGER signaling. An inset in the AlzPathway part of Figure 4 illustrates details at a higher zoom level.

### 3.4 Connectivity of interactions

**FIGURE 5:**
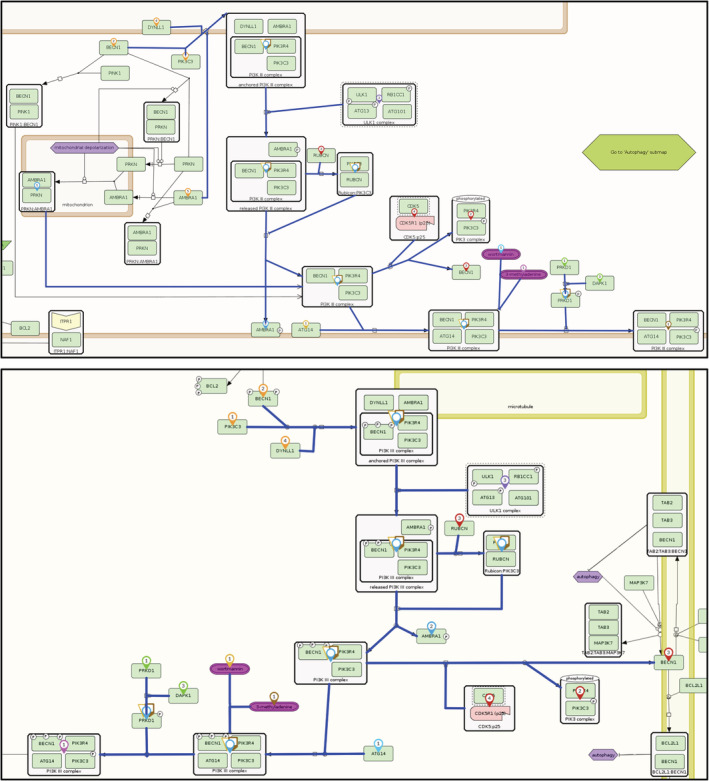
Visualization of protein interaction clusters having high similarity between two disease maps. Two clusters of autophagy‐related mechanisms are shown, with the top part from the Parkinson's disease map, the bottom from the Aging map. These results are selected from a global comparison between the Maps as an example. In this Figure, similar reactions are highlighted in blue, their elements are marked with colored pins. Although the layouts of these reaction clusters are different, key components are similar, supporting reproducible and fast identification of common mechanisms.

Next, we investigated which areas of disease maps may be similar to each other. For this purpose, we used the list of previously calculated similarities between the interactions of the three disease maps. We then constructed a bipartite graph from each disease map, where “reactant” and “modifier” type map elements are nodes that create input edges to nodes representing “reactions”, and “reaction” nodes create input edges to nodes representing “product” type elements. These bipartite graphs were filtered to contain only the reactions identified in Section 3.3. In such reduced graphs, we then identified connected components. As we aimed to identify entire areas in disease maps, we only retained connected components containing three or more “reaction” type nodes. Such an approach automates the process of visual examination of similar areas in different diagrams and identifies mechanisms of interest composed of multiple similar reactions. Matching connected components in compared disease maps are listed in Table 4. Also, Figure 5 illustrates an example of a similar area in different maps. As the MINERVA Platform allows exporting such diagram components into standardized formats, we provide a set of overlapping diagram areas between the Parkinson's disease map and the Aging map as editable files (Ostaszewski, 2022) in the standard CellDesigner format (Kitano et al., 2005).

**TABLE 4:** Similarity of interactions between diagrams in the selected disease maps.

Comparison	Nb of similar interactions	Nb of connected components	Main areas/pathways
Aging versus PD	342	21	Autophagy (Lo Surdo et al., 2018) Cell death Mitophagy (Lo Surdo et al., 2018) Mitochondrial respiration (Mazein et al., 2018) Mitochondrial unfolded protein response MTOR‐AMPK signaling Inflammation (UniProt Consortium, 2021) ROS‐dependent NFE2L2 activity PPARGC1A activity Ubiquitin Proteasome System
AD versus PD	43	2	Autophagy Inflammation/Cytoskeleton homeostasis
Aging versus AD	34	2	Autophagy Inflammation/Cytoskeleton homeostasis

## 4 DISCUSSION

With the increasing number of community‐provided systems biology diagrams, it is important to develop workflows for efficient exploration and comparison of such resources. As such resources are published in a decentralized manner, they require a different approach than pathway databases. Here, we introduce the MINERVA Net registry, a web service allowing individual users to share minimal information about systems biology diagrams hosted on different MINERVA Platform instances. The content can be curated by authors independently and APIs offered by MINERVA Net and MINERVA Platform support construction of reproducible workflows, which can be run regularly providing up‐to‐date results. This setup allows investigation of common and disease‐specific molecular mechanisms, including the role played by different proteins.

Here, we demonstrate the use of the MINERVA Net repository by comparing the contents of three disease maps: Parkinson's disease map, Aging map and AlzPathway. In particular, we focused on proteins in these resources, represented by their stable UniProt identifiers. Using dedicated functionalities of the minervar R package, we compared the protein content across these disease maps, calculated similarities between complex protein interactions, and identified areas in the map functionally connecting these similar interactions. Interestingly, a comparison of unconnected identifiers leads to different similarity results than comparison of networks underlying disease maps. As MINERVA Net and MINERVA Platform APIs offer access to rich information about hosted systems biology diagrams, we believe they can support the use of complex similarity measures.

The analysis presented in this article has certain shortcomings. First, similarity results, especially those based on comparing interactions, depend heavily on the stylistic choices of a curator constructing a given diagram. This may be the reason for the high similarity of PD map and Aging map. To overcome this challenge, better approaches to content comparison have to be proposed. One of such metrics could be Euclidean distance between map elements (Ostaszewski et al., 2018), information also available in the MINERVA Platform. Second, focusing on UniProt identifiers provides an excellent common denominator across diagrams, but this way information on protein state and posttranslational modifications is dropped, which could otherwise support more accurate comparisons.

The MINERVA Net repository allows efficient and reproducible comparison of disease maps and similar systems biology diagrams. One of the next steps in the platform development is to ensure broad participation of content creators by sharing their work via the repository. This can be addressed by actively reaching out to the users via domain‐relevant meetings, and by providing training materials explaining this new feature of the MINERVA ecosystem. This will preserve the content and ensure its reuse after the map curation is over, for example, for AlzPathway. Active community engagement can lead to establishing consistent guidelines for diagram building, reducing the variability of design choices, and improving the accuracy of compared and shared content (Hanspers et al., 2021). Another avenue for development is improvement of the comparison metrics. Detailed information on protein state and posttranslational modifications are available via the MINERVA API if provided by the curator. This gives an opportunity to design more sophisticated comparison metrics, possibly including omics data for more contextualized comparisons.

## AUTHOR CONTRIBUTIONS

**Piotr Gawron:** Conceptualization (equal); investigation (supporting); project administration (supporting); software (lead); writing – review and editing (supporting). **Ewa Smula:** Investigation (supporting); project administration (supporting); software (supporting); writing – review and editing (supporting). **Reinhard Schneider:** Conceptualization (supporting); funding acquisition (lead); investigation (supporting); project administration (supporting); resources (lead); supervision (supporting); writing – review and editing (supporting). **Marek Ostaszewski:** Conceptualization (equal); investigation (lead); project administration (lead); supervision (lead); writing – original draft (lead).

## Supporting information


**Appendix S1.** Supplementary materials

## Data Availability

The data that support this study are available in the MINERVA Net repository at https://minerva-net.lcsb.uni.lu, last accessed on 12 September 2022. These data refer to the following resources available in the public domain: Parkinson's disease map (pdmap.uni.lu), Ageing map (progeria.uni.lu) and AlzPathway (http://www.alzpathway.org/AlzPathway.html, https://pathwaylab.elixir‐luxembourg.org/minerva/index.xhtml?id=alzpath_Nov18). The code for the analysis is available at https://gitlab.lcsb.uni.lu/minerva/api-scripts/
